# Prognostic impact of thrombocytosis in gastric cancer—A retrospective study

**DOI:** 10.1097/j.pbj.0000000000000247

**Published:** 2024-03-08

**Authors:** Bárbara Neto Castro, Catarina Costa, Daniel Martins, Andreia Amado, Mariana Santos, Susana Graça, Amélia Tavares, António Ferreira, Fernando Viveiros, Sílvio Vale, Manuel Oliveira

**Affiliations:** General Surgery Department, Centro Hospitalar Vila Nova de Gaia/Espinho, Vila Nova de Gaia, Portugal

**Keywords:** gastric cancer, thrombocytosis

## Abstract

**BACKGROUND:**

Solid tumors are a common cause of secondary thrombocytosis, which has been identified as a prognostic factor in various cancers. However, the impact of thrombocytosis on the prognosis of gastric cancer is not yet well defined. The aim of this study was to assess the prevalence and prognostic value of thrombocytosis in patients with gastric cancer.

**METHODS:**

This was a retrospective study of patients with gastric carcinoma treated surgically, with curative intent, in our hospital, Centro Hospitalar Vila Nova de Gaia/Espinho, between January 2009 and December 2019. Clinical files were consulted and clinicopathological characteristics were analyzed.

**RESULTS:**

In the present sample (n = 352), the prevalence of pretreatment thrombocytosis was 16.5%. Thrombocytosis was associated with more advanced T stage, greater number of metastatic nodes, and more frequent lymphatic and venous permeation. The presence of thrombocytosis had a negative impact on disease-free survival (hazard ratio [HR] 3.54, 95% confidence interval [CI] 2.35–5.33, *P* < .001) and overall survival (HR 4.45, 95% CI 2.95–6.71, *P* < .001).

**CONCLUSIONS:**

The presence of pretreatment thrombocytosis had a negative impact on overall survival and disease-free survival and thus could be used as an independent prognostic factor.

## Introduction

Gastric cancer is a worldwide leading cause of cancer-related death. The 5-year survival is lower than 20% and remains under the expectation because patients after surgery have a high risk of local and distant recurrence.^1,2^ Many factors influence the prognosis of gastric cancer, including the type of cancer, stage at diagnosis, age, sex, race, overall health, and lifestyle.^2^

Solid tumors are a common cause of secondary thrombocytosis, which has been identified as a prognostic factor in various cancers, such as renal cell carcinoma, colorectal cancer, and cervical cancer.^3^

Several studies suggest a relationship between the coagulation system, platelet function, and hematogenous tumor spread.^4^ Platelets are considered a *tumor promoter* because they have multiple ways to contribute to tumor growth, survival, and metastasis. The exact pathophysiological mechanisms are still not completely understood. The increase in platelet count is thought to be due to the release of thrombopoietic cytokines by tumor cells. Platelets then facilitate metastasis by protecting circulating tumor cells from natural killer cells and by supporting seeping of the circulating tumor cells.^5^ Furthermore, platelets also store different growth factors that promote tumor growth, angiogenesis, and tumor cell migration, which induce more thrombopoiesis, leading to a vicious cycle.^4^

The impact of thrombocytosis on the prognosis of gastric cancer is not yet well defined. The prevalence of thrombocytosis in patients with gastric cancer ranges from 6.4% to 20.4%.^6^ The result of a recent systematic review and meta-analysis, in which 10 studies were included, comprising 8166 patients with gastric cancer, showed that patients with thrombocytosis had significant worse overall survival than those with normal platelet count and were associated with advanced clinical stage, deeper tumor invasion, and higher risk of recurrence.^1^

The association between a high platelet count at the time of diagnosis and worse prognosis has been extensively studied in gastrointestinal neoplasms.^4^ However, the few reports in this subject are not unanimous, especially regarding to gastric cancer. Therefore, the aim of this study was to assess the prevalence and prognostic value of thrombocytosis in patients with gastric cancer.

## Methods

All data collection was performed under strict ethical and confidentiality procedures. The manuscript is in accordance with the ethical standards of the institution and with the Declaration of Helsinki.

Medical records of patients with gastric carcinoma treated surgically, with curative intent, at the Department of Surgery, in our hospital, Centro Hospitalar Vila Nova de Gaia/Espinho, between January 2009 and December 2019 were included in this retrospective study. Patients with a diagnosis of gastric carcinoma not undergoing surgery or undergoing palliative surgery, patients with R1 or R2 resections, and patients with metastasis at the time of surgery were excluded. Based on eligibility and exclusion criteria, 352 patients were enrolled in our study. Clinical files were consulted and clinicopathological characteristics were analyzed.

Blood platelet count was evaluated at the time of diagnosis and before any treatment. Thrombocytosis was defined as a platelet count >400 × 10^3^/μL.

To correlate categorical variables, chi-squared and Fisher exact tests were applied. To correlate continuous variables, Mann-Whitney test was applied. Disease free-survival and overall survival were estimated by the Kaplan-Meier method, and the log-rank test was used to determine univariate significance. The Cox regression was applied in univariate and multivariate prognosis analyses. In the multivariate analysis, one only considered the variables with prognostic value according to the univariate analyses. Significance was defined as *P* < .05. Statistical analysis was performed using IBM SPSS Statistics 26.0.

## Results

In our sample, the mean age was 68 ± 12 years, ranging from 29 to 88 years, and 52% of patients were female.

In this study, the mean platelet count was 276.10 ± 108.60 × 10^3^/μL, ranging from 55 to 684 × 10^3^/μL. All patients were divided in two groups: thrombocytosis group (58 patients) and thrombocytosis absent group (294 patients). The incidence of thrombocytosis in our study was 16.5%.

Clinicopathological characteristics of groups with and without thrombocytosis are presented in Table 1. Patients with thrombocytosis were found to have a more advanced T stage (*P* = .002), greater number of metastatic nodes (*P* < .001), and a more frequent presence of lymphatic and venous permeation (*P* < .001).

**Table 1:** Clinicopathological characteristics of groups with and without thrombocytosis

Characteristic	Thrombocytosis Present (n = 58)	Thrombocytosis Absent (n = 294)	*P*
Sex			.771
M	27 (47%)	143 (49%)	
F	31 (53%)	151 (51%)	
Age (years)			.606
<45	2 (3%)	16 (5%)	
45–64	18 (31%)	108 (37%)	
>65	38 (66%)	170 (58%)	
Surgery			.407
TG Y Roux	24 (41%)	129 (44%)	
STG Y Roux	27 (47%)	140 (48%)	
STG BII	7 (12%)	19 (6%)	
Totalization	0 (0%)	6 (2%)	
pT			.002
is	0 (0%)	4 (1%)	
1a	2 (3%)	44 (15%)	
1b	4 (7%)	51 (17%)	
2	9 (16%)	54 (18%)	
3	28 (48%)	108 (37%)	
4a	13 (22%)	31 (11%)	
4b	2 (3%)	2 (1%)	
pN			<.001
N0	12 (21%)	149 (51%)	
N1	10 (17%)	51 (17%)	
N2	13 (22%)	44 (15%)	
N3a	15 (26%)	36 (12%)	
N3b	8 (14%)	14 (5%)	
TNM			<.001
0	0 (0%)	4 (1%)	
IA	6 (10%)	72 (24%)	
IB	4 (7%)	46 (16%)	
IIA	2 (3%)	50 (17%)	
IIB	7 (12%)	34 (12%)	
IIIA	14 (24%)	42 (14%)	
IIIB	16 (28%)	36 (12%)	
IIIC	9 (16%)	10 (3%)	
G (n = 334)			.108
1	4 (7%)	47 (17%)	
2	20 (34%)	98 (36%)	
3	34 (59%)	131 (47%)	
Lymphatic permeation (n = 343)			<.001
Present	49 (88%)	182 (64%)	
Absent	7 (13%)	105 (37%)	
Venous permeation (n = 340)			<.001
Present	43 (77%)	104 (36%)	
Absent	13 (23%)	180 (63%)	
Neoadjuvant treatment			.781
Yes	9 (16%)	50 (17%)	
No	49 (84%)	244 (83%)	
Adjuvant treatment			.186
Yes	25 (43%)	100 (34%)	
No	33 (57%)	194 (66%)	
Recurrence			<.001
Yes	37 (64%)	66 (22%)	
No	21 (36%)	228 (78%)	

BII, Bilroth II; F, female; G, grade of differentiation; M, male; STG, subtotal gastrectomy; TG, total gastrectomy; TNM, tumor, nodes, metastasis.

**Figure 1.**
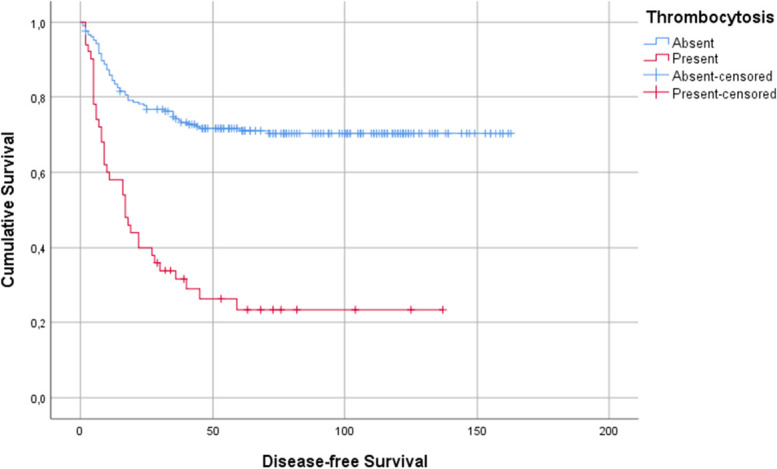
Impact of thrombocytosis on disease-free survival of patients with gastric cancer.

Using Kaplan-Meier curves, we ascertained that the presence of pretreatment thrombocytosis had a negative impact on disease-free survival, which was statistically significant (log-rank test *P* < .001), as shown in Fig. 1. The median disease-free survival in the group without thrombocytosis was 115 ± 5 months compared with 44 ± 8 months in the thrombocytosis group. One-year, 3-year, and 5-year disease-free survival were lower in the thrombocytosis group, as shown in Table 2.

**Table 2:** Disease-free survival and overall survival of groups with and without thrombocytosis

Disease-free survival	All patients	Thrombocytosis absent	Thrombocytosis present
1 year	80.5%	85.5%	59.2%
3 years	64.0%	72,0%	30.3%
5 years	60.6%	69.4%	22.5%

Overall survival	All patients	Thrombocytosis absent	Thrombocytosis present
1 year	94.4 %	94.5%	78.3%
3 years	76.3%	83.3%	42.0%
5 years	66.1%	74.5%	30.4 %

Univariate Cox regression showed that the presence of thrombocytosis had negative association with disease free-survival (*P* < .001 and hazard ratio [HR] 3.54) and that those patients had a recurrence risk about 3.5 times higher than patients without thrombocytosis (Table 3). In the multivariate analysis, the presence of thrombocytosis at the time of diagnosis remained an independent prognostic factor (*P* = .007 and HR 1.823), as shown in Table 3.

**Table 3:** Univariate and multivariate prognostic analyses of gastric cancers (disease-free survival)

Variable	Univariate analysis			Multivariate analysis		
	*P*	HR	IC 95%	*P*	HR	IC 95%
Sex	.212	0.781	0.530–1.151	Excluded		
Age	.296	1.009	0.992–1.027	Excluded		
Thrombocytosis	<.001	3.541	2.356–5.332	.007	1.823	1.176–2.827
pT	<.001	14.909	5.480–40.558	.004	8.434	1.970–36.102
pN	<.001	4.991	3.028–8.227	.007	2.216	1.248–3.936
G	.001	5.612	2.063–15.265	.237	2.372	0.566–9.936
Venous permeation	<.001	4.428	2.847–6.888	.055	1.658	0.989–2.779
Lymphatic permeation	<.001	5.339	2.851–9.997	.180	1.719	0.778–3.794
Neoadjuvant treatment	.490	0.836	0.502–1.391	Excluded		
Adjuvant treatment	.424	1.171	0.795–1.724	Excluded		

G, grade of differentiation; pM, pathological nodes staging, metastasis; pT, pathological tumor staging.

**Figure 2.**
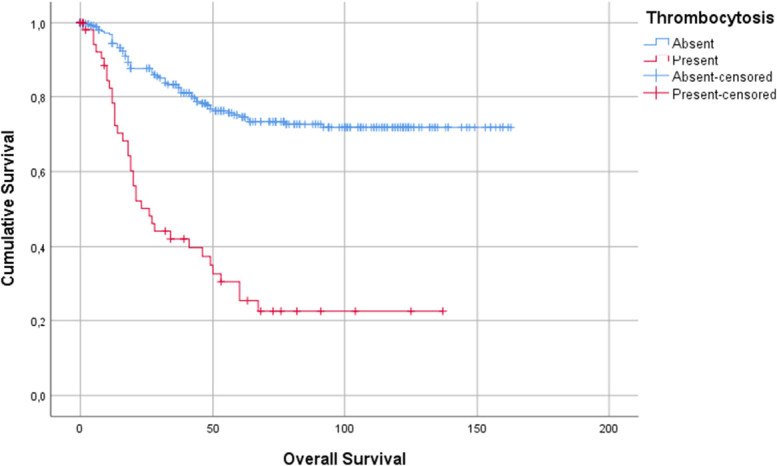
Impact of thrombocytosis on overall survival of patients with gastric cancer.

Regarding overall survival, thrombocytosis also had a negative and statistically significant association, as evident in the Kaplan-Meier curves, shown in Fig. 2. The median overall survival in the group without thrombocytosis was 126 ± 4 months compared with 50 ± 7 months in the thrombocytosis group. One-year, 3-year, and 5-year overall survival were lower in the thrombocytosis group, as shown in Table 2.

Univariate Cox regression showed that the presence of thrombocytosis negatively related with overall survival (*P* < .001 and HR 4.45) and that these patients had a risk of death from cancer 4.5 times higher than patients without thrombocytosis (Table 4). In the multivariate analysis, the presence of thrombocytosis at the time of diagnosis remained an independent prognostic factor (*P* < .001 and HR 2.248), as shown in Table 4.

**Table 4:** Univariate and multivariate prognostic analyses of gastric cancers (overall survival)

Variable	Univariate analysis			Multivariate analysis		
	*P*	HR	IC 95%	*P*	HR	IC 95%
Sex	.291	0.809	0.546–1.1 99	Excluded		
Age	.742	1.003	0.986–1.020	Excluded		
Thrombocytosis	<.001	4.45	2.956–6.712	<.001	2.248	1.440–3.512
pT	<.001	14.671	5.391–39.923	.006	7.799	1.823–33.368
pN	<.001	6.096	3.610–10.291	.001	2.831	1.542–5.201
G	.001	5.801	2.131–15.789	.264	2.272	0.538–9.596
Venous permeation	<.001	4.650	2.982–7.250	.027	1.791	1.067–3.009
Lymphatic permeation	<.001	4,835	2,580–9,062	.463	1.340	0.613–2.935
Neoadjuvant treatment	.917	1.028	0.609–1.735	Excluded		
Adjuvant treatment	.274	1.246	0.840–1.849	Excluded		

G, grade of differentiation; pM, pathological nodes staging, metastasis; pT, pathological tumor staging.

## Discussion

An elevated platelet count is commonly seen in many cancers and is associated with a worse prognosis. In our study, the mean level of platelet count was 276.10 ± 108.60 × 10^3^/μL, and 16.5% of patients with gastric cancer had thrombocytosis, which was consistent with previous reports.^6^ This study demonstrated that thrombocytosis correlated with clinical features, such as advanced stage and a more frequent lymphatic and venous permeation. We have also found that thrombocytosis associated with increased risk of recurrence, but not with tumor differentiation, which could be explained by the effect of platelets on promoting tumor metastasis but not influencing tumor biology, as highlighted by other authors.^1^

We also found that thrombocytosis was associated with worse overall survival and disease-free survival, which is in line with recent evidence.^1^ As expected, the T stage and N stage also had a statistically significant negative impact on survival.

The largest systematic review and meta-analysis published, included 10 studies, four of them prospective in nature, involving 8166 patients with gastric cancer. Our study is in line with their conclusions that patients with thrombocytosis had significant worse overall survival and higher risk of recurrence.

Platelet count is easily obtained by routine blood test and is inexpensive, so it would be of great value as a prognostic tool. It was observed in some malignancies a rise in platelet count when recurrence occurs, which raises the possibility of also using thrombocytosis during follow-up.^4^

Our study has potential limitations. First, this was a retrospective, single-center study. We also did not assess whether patients were receiving any antithrombotic therapy or whether they had a history of thrombotic events before cancer. We also were unable to explain the lack of impact of neoadjuvant and adjuvant therapies, but we did not meticulously analyze whether patients completed chemotherapy regimens.

In the future, large scale multicenter prospective studies will be needed to validate thrombocytosis as an independent prognostic factor and to understand whether thrombocytosis may be another factor to weigh in the decision of neoadjuvant and adjuvant treatments. We may also be facing another therapeutic target as there are already some studies with antiplatelet agents working as antitumor agents.^7^ Additional investigation is needed to understand whether therapy targeting platelets will be a successful oncologic treatment.

In conclusion, pretreatment thrombocytosis is a useful predictor of overall survival and disease-free survival in patients with gastric cancer and thus could be used as an independent prognostic factor.

## Acknowledgments

Assistance with the study: none.

Financial support and sponsorship: none.

Presentation: This article was previously presented as a meeting abstract at the SKY Meeting on November 19 and 20, 2022.

## Author contributions

All authors contributed to the study conception and design. The first draft of the manuscript was written by Bárbara Neto Castro, and all authors commented on previous versions of the manuscript. All authors read and approved the final manuscript.

## Conflicts of interest

The authors declare no conflicts of interest.
